# Fabrication of Cocatalyst NiO-Modified BiVO_4_ Composites for Enhanced Photoelectrochemical Performances

**DOI:** 10.3389/fchem.2022.864143

**Published:** 2022-06-02

**Authors:** Zhi-Qiang Wang, HongJun Wang

**Affiliations:** ^1^ School of Materials Science and Engineering, North University of China, Taiyuan, China; ^2^ School of Materials Science and Engineering, Jilin University, Changchun, China

**Keywords:** bismuth vanadate, nickel oxide, photoelectrochemical, cocatalyst, water oxidation

## Abstract

In this work, NiO modified BiVO_4_ (BiVO_4_/NiO) nanocomposite was synthesized using hydrothermal and calcination method. The composite of BiVO_4_/NiO, further employed as a low-overpotential photoanode, was consisted of BiVO_4_ nanoparticles and NiO nanosheets, in which the BiVO_4_ nanoelectrode served as the matrix for the attachment of NiO nanosheets. Photoelectrochemical (PEC) tests show that BiVO_4_/NiO displayed improved PEC performance compared with pure BiVO_4_. The BiVO_4_/NiO photoanode delivers a photocurrent density of 1.2 mA/cm^2^ at 1.23 V vs. RHE in a Na_2_SO_4_ electrolyte under an AM 1.5G solar simulator, which is 0.3 mA/cm^2^ higher than pure BiVO_4_ photoanode. Meanwhile, the onset potential also generates a 350 mV cathodic shift. The enhanced performance of the BiVO_4_/NiO nanocomposite is attributed to NiO unique lamellar structure capable of providing a large number of active sites. Measurements of electrochemical impedance spectra (EIS) and the incident photon-to-current efficiency (IPCE) illustrate that the enhanced PEC activities are ascribed to the improved charge carrier separation/transport and the promoted water oxidation kinetics furnished by the decoration of NiO cocatalyst.

## Introduction

Due to the excessive consumption of fossil energy that results in severe environmental pollution worldwide, the development of clean and sustainable energy technologies has received increasing attention. (Iwase et al., 2011; Guo et al., 2014; Zeng et al., 2021a) Clean hydrogen production is seen as a promising strategy, capable of simutaneously addressing climate change and environmental issues related to fossil fuel combustion. (Wang L. et al., 2018; Fukuzumi et al., 2018; Kim et al., 2018) Photoelectrochemical (PEC) water splitting, capable of directly converting solar energy into chemical energy, is considered a promising technology for converting solar energy into stable chemical energy, thus becoming attractive for reducing pollution associated with energy production. (Zhang J. et al., 2016; Wang and Wang, 2018; Weng et al., 2018; Zeng et al., 2019) In the PEC system, the photoanode acts the role of reaction sites for effective oxygen evolution. (Liu et al., 2014; Roger et al., 2017) In conclusion, the development of efficient photoanode materials is of great significance for constructing a practical PEC water splitting system. Various semiconductors including TiO_2_ (Crake et al., 2017; Zeng et al., 2020), ZnO (Han et al., 2015; Hong et al., 2015), *α*-Fe_2_O_3_ (Dotan et al., 2011; Huang et al., 2016), WO_3_ (Huang et al., 2017; Li et al., 2018), BiVO_4_ (Wang et al., 2017a; Wang Q. et al., 2018) and BiOBr (Wang Z.-Q. et al., 2020) etc. have been developed as photocatalytic materials. Among them, scheelite-monoclinic bismuth vanadate (BiVO_4_) has been widely studied for PEC water splitting owing to its relatively narrow band gap of 2.4 eV for visible-light absorption, as well as an appropriate band position for effective water oxidation and high stablility. (Malathi et al., 2018) However, the application of BiVO_4_ is still restricted by its inherent defects such as low charge transport (Zhou et al., 2018), high charge recombination (Zhang Y. et al., 2016) and slow poor water oxidation kinetics (Zhai et al., 2017). The photocurrent density of the pure BiVO_4_ is obviously lower than its theoretical value of 7.5 mA/cm^2^ (Xu et al., 2014; Chen, 2015) To overcome these issues, transition metal-based catalysts used as cocatalysts are one of the effective ways to improve the PEC water splitting performance.

Transition metal-based materials, especially (Co., Ni, Fe)-based materials, comparable to precious metals because of their low cost and advanced catalytic performance, are considered to be the most promising OER catalysts. (Zhang Q. et al., 2016; Kuang et al., 2016; Wang Z. et al., 2020)The combination of the OER catalyst and the semiconductor light absorber can not only improve the PEC activity by providing an interface reaction active site that reduces overpotential, but also improve the PEC stability by rapidly consuming photo-generated carriers of semiconductor materials against electrolytes (Zhou et al., 2015). (Trotochaud et al., 2014; Zeng et al., 2021b) Recently, Dai (Kenney et al., 2013; Li et al., 2017) and colleagues deposited an ultra-thin nickel film on the n-type silicon substrate as a physical protective layer. It was found that a 2 nm nickel film played a crucial role in the sustainability of n-type silicon photoanodes in the solar-driven water oxidation process. The ultra-thin nickel was used as a protective layer and a passivation layer, and the natural NiO_x_ formed during the test was employed as an OER promoter. The NiOx/Ni/n-Si photoanode can work under constant photocurrent of 10 mA/cm^2^, and still have excellent stability after water oxidation of 80 h. In addition, Lewis (Zhou et al., 2019) and his colleagues introduced an ultra-thin CoO_x_ film as an intermediate layer between NiO_x_ layer and n-Si to enhance the interaction between co-catalysts/semiconductors. It was found that further passivation of the CoO_x_ layer on the n-Si surface can change the initiation. The potential was more negative than NiO_x_/SiO_x_/n-Si photoanode. NiOx/CoO_x_/SiO_x_/n-Si showed the most negative flat band position, which was related to the barrier height in the semiconductor, and therefore highly improved the separation and collection of charge carriers. The above results indicate that the combination of NiO and other favorable semiconductors can remarkably reduce the overpotential of the photoelectrode.

In this study, a BiVO_4_ photoanode, acting the role of photoelectrocatalysis substrate, is synthesised by a electrodeposition-calcination method. On the other hand, a nanosheet structured NiO, playing the performance of the water oxidation cocatalyst to combine with the BiVO_4_ photoanode and thus improve the PEC performance, is preprared via a hydrothermal-calcination. Under AM 1.5G sunlight, the BiVO_4_/NiO film produced a relatively high photocurrent density of 1.2 mA/cm^2^ at 1.23 V vs. RHE, much higher than that of the pure BiVO_4_ film. More importantly, the onset potential is negatively shifted by 350 mV relative to pure BiVO_4_. The special structure of NiO is believed to be beneficial to absorb more incident photons through the light-harvesting effect, thereby enhancing the separation and transport of photo-induced charge carriers. Furthermore, the deposition of NiO cocatalyst on the surface of the BiVO_4_ photoanode significantly promotes the water oxidation kinetics.

## Experimental

### Chemicals

Bi(NO_3_)_3_·5H_2_O (Sinopharm Chemical Reagent Co., Ltd., 99.0%), potassium iodide, ethylene glycol are purchased from chemical reagent co, Ltd. P-benzoquinone (Tianjin Institute of Fine Chemicals, 99.0%). Ni(NO_3_)_2_·6H_2_O (Sinopharm Chemical Reagent Co., Ltd., 99.0%), hexamethylenetetramine (HMTA, Chengdu Cologne Chemicals Co., Ltd., 99.0%), Anhydrous ethanol was purchased from Sinopharm Chemical Reagent Co., Ltd. All aqueous solutions were prepared with deionized water.

### Preparation of BiVO_4_

The preparation of BiVO_4_ was synthesized with reference to previous reported work. Systematically, 50 ml of 0.4 M KI solution was first adjusted to pH 1.7 with 1 M HNO_3_, and then 5 mmol Bi(NO_3_)_3_·5H_2_O was added with rapid stirring until dissolved, resulting in an orange-red mixed solution. Then 20 ml of ethanol containing 4.6 mmol of 1,4-benzoquinone was slowly added dropwise to the above solution and stirred for several tens of minutes. Next, BiOI nanosheets were synthesized in a three-electrode system by electrodeposition. Among them, the platinum electrode was used as the counter electrode, the clean FTO glass was used as the working electrode, and the Ag/AgCl (3.5 M KCl) electrode was used as the reference electrode. Cyclic voltammetry (CV) was used for electrodeposition, and the resulting membrane was rinsed with distilled water to obtain a clean BiOI membrane. Immediately after, 150 μl of 0.2 M vanadyl acetylacetonate (VO(acac)_2_) DMSO solution was dropped onto the above BiOI nanosheets. Calcined at 450°C for 2 h at a ramp rate of 2°C/min. The cooled membrane was washed with 1 M NaOH solution to remove excess V_2_O_5_ from the BiVO_4_ electrode.

### Preparation of BiVO_4_/NiO Photoanode

The BiVO_4_/NiO photoanode was prepared by a hydrothermal method. The configuration takes 60 ml of solution in which the volume ratio of deionized water and ethanol solution is 2:1. After ultrasonically mixed uniformly, 1.5 mmol Ni(NO_3_)_2_·6H_2_O and 6 mmol HMTA were added thereto, and stirred until dissolved. Then 20 ml of the above mixture was added to a PTFE-lined stainless steel autoclave (100 ml). And the as-prepared BiVO_4_ photoanode was placed obliquely with the conductive side facing upwards, heated at 90°C for 4 h. The photoanode was washed three times with water and ethanol and dried at 60°C for 12 h. Finally, the BiVO_4_/NiO photoanode was obtained after calcination in air with a heating rate of 2°C/min at 300°C for 2 h.

### Characterizations

The microscopic morphology of the samples was characterized by scanning electron microscopy (SEM, JSM-6701E). The crystal structure of the as-prepared photoanode was measured by X-ray diffraction (XRD) tests on the X-ray diffractometer (D/MAX-2200/PC). UV-Vis diffuse reflectance spectroscopy was used to investigate the response of the prepared photoelectrode to visible light on a UV-3100 spectrometer.

### PEC Characterizations

The photoelectrochemical tests of the as-prepared photoanodes were carried out on a CHI 660D electrochemical workstation. A three-electrode system was used, in which a platinum sheet, Ag/AgCl (3.5 M KCl), and the samples were the counter electrode, the reference electrode and the working electrode, respectively. The electrolyte is 0.5 M Na_2_SO_4_ solution (pH = 6.86). All tests were performed with FTO backside irradiation at room temperature. The scan rate for linear sweep voltammetry (LSV) was 10 mV s^−1^. The light intensity was calibrated to 100 mW/cm^2^ with an optical power meter. And the incident photon current efficiency (IPCE) was measured using a 300 W xenon lamp with a monochromator in 0.5 M Na_2_SO_4_ electrolyte at 1.23 V vs. RHE. The photogenerated photocurrent $\left(J_{abs}\right)$ undergo two major losses of charge carriers recombination in bulk and at interface. Hence the measured photocurrent during water oxidation $\left(J_{H_{2}O}\right)$ is expressed as follows:

$${\text{J}}_{{\text{H}}_{2}\text{O}}={\text{J}}_{\text{a}\text{b}\text{s}}\times \eta_{\text{i}\text{n}\text{j}}\times \eta_{\text{s}\text{e}\text{p}}$$

where J_abs_ is obtained by integrating the distribution of solar power density P(λ) with light absorption α(λ) of the photoanode as equation:

$${\text{J}}_{\text{a}\text{b}\text{s}}=\text{e}\overset{\lambda_{\text{a}\text{b}\text{s}}}{\underset{0}{\int }}\alpha \left(\lambda \right)\frac{\text{P}\left(\lambda \right)}{\text{h}\upsilon }\text{d}\lambda$$

where $\alpha \left(\lambda \right)=1-10^{-A}$ , A is the absorbance according to the UV-vis spectrum, P(λ) = the distribution of solar power density.

The photocurrent during sodium sulfite oxidation was measured $\left(J_{SO_{3}^{2-}}\right)$ in order to calculate charge separation efficiencies. Since the interface charge separation efficiency of sodium sulfite oxidation is almost 100% $\left(\eta_{\text{sep },\;SO_{3}^{2-}}=1\right)$ , the charge separation efficiencies can be calculated as follows:

$$\eta_{\text{i}\text{n}\text{j}}={\text{J}}_{\text{S}{\text{O}}_{3}^{2-}}/{\text{J}}_{\text{a}\text{b}\text{s}}\;$$

$$\eta_{\text{s}\text{e}\text{p}}={\text{J}}_{{\text{H}}_{2}\text{O}}/{\text{J}}_{\text{S}{\text{O}}_{3}^{2-}}\;$$

## Results and Discussion

### Characterization of the BiVO_4_/NiO Composite Photoanode

**FIGURE 1:**
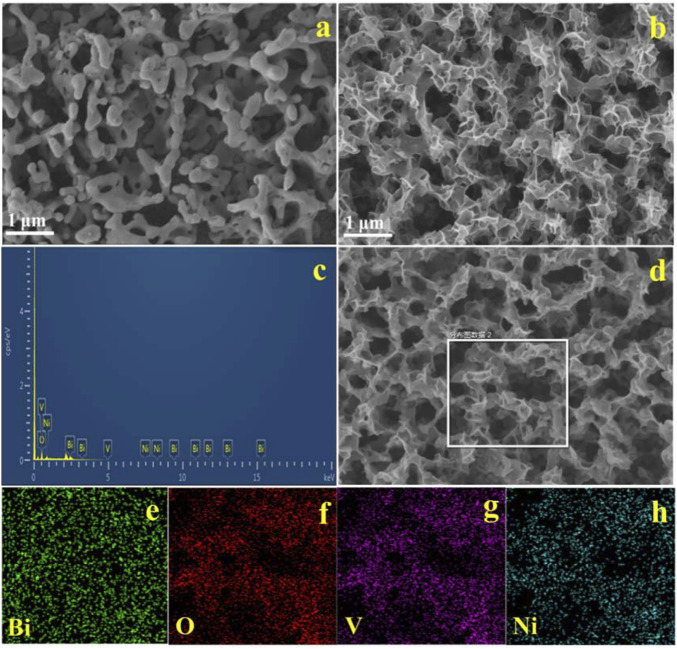
SEM images of the typical samples: **(A)** BiVO_4_, **(B)** BiVO_4_/NiO, EDS pattern **(C)** and the EDS elemental mapping **(D–H)** of as prepared BiVO_4_/NiO photoanode.

The morphology and elemental compositions of the synthesized BiVO_4_, BiVO_4_/NiO composite photoanode were studied with SEM and energy dispersive spectroscopy (EDS) in Figures 1A–H. Figure 1A exhibits the SEM image of nanoporous BiVO_4_ film. Figure 1B shows the SEM image of BiVO_4_/NiO. NiO nanosheets with size distribution about 5–10 nm are fairly continuous and uniformly loaded on the surface of BiVO_4_ in large area. The elemental composition and content of BiVO_4_/NiO anodes were further investigated by EDS. Figure 1C shows the EDS pattern of BiVO_4_/NiO photoanode, the weight percentage of elements present in the BiVO_4_/NiO photoanode composites are 39.4%, 31.1%, 18.6% and 10.09% for Bi, O, V and Ni, respectively. No other elements or impurities are found. The elemental composition and distribution in the BiVO_4_/NiO photoanode were further observed by EDS elemental mapping (Figures 1D–H **)**. The results clarify that the elements of Bi, O, V and Ni are present and uniformly distributed in the BiVO_4_/NiO photoanode.

**FIGURE 2:**
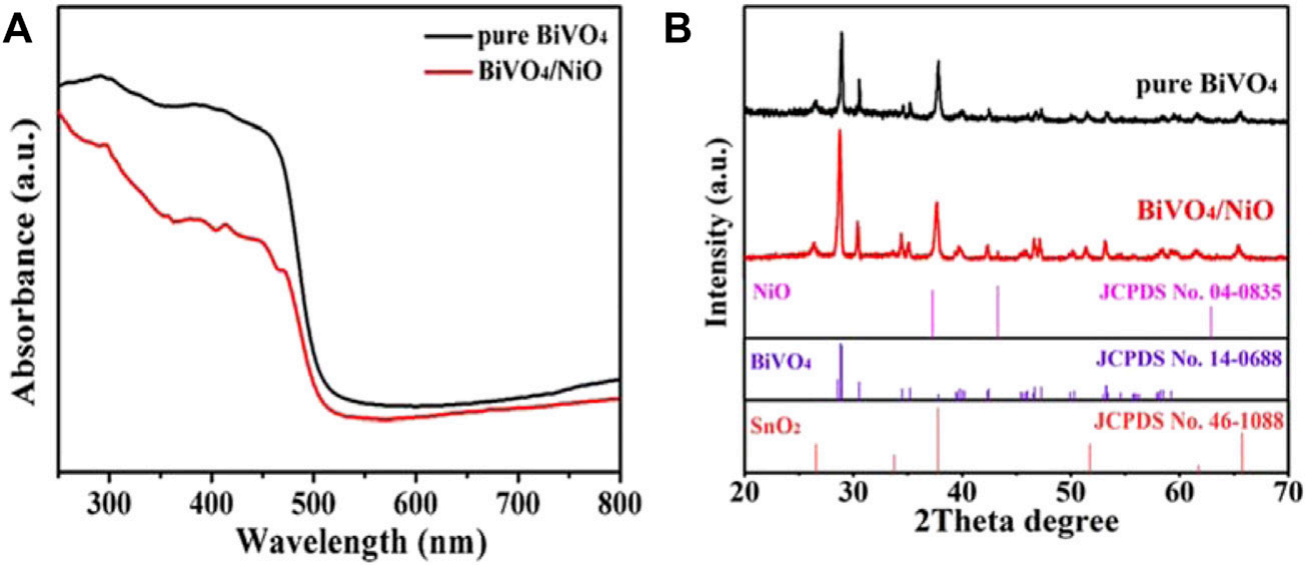
**(A)** UV−Vis diffuse reflectance spectra of BiVO_4_ and BiVO_4_/NiO. **(B)** XRD patterns of the BiVO_4_ and BiVO_4_/NiO photoanodes.

The optical absorption properties of pure BiVO_4_ and BiVO_4_/NiO films were investigated by UV-Vis diffuse reflectance spectroscopy. It can be seen that BiVO_4_ and BiVO_4_/NiO show good light absorption properties around 500 nm (Figure 2A), corresponding to a band gap of 2.4 eV. Notably, the BiVO_4_/NiO sample shows almost the same absorption edge as bare BiVO_4_ due to the blocking by the thicker BiVO_4_ layer, indicating that coating of NiO almost rarely affects light absorption of BiVO_4_/NiO. But the BiVO_4_ photocathode loaded with NiO co-catalyst shows the relatively low light absorption capacity. The reason is primarily ascribed to the poor optical transparency of the nickel based co-catalyst. (Zhou et al., 2020)

The XRD pattern characterization reveals that the product is composed of three kinds of materials with distinct crystal structures. Figure 2B displays the XRD patterns of BiVO_4_ and BiVO_4_/NiO nanocomposites. The XRD diffraction peaks of BiVO_4_ and BiVO_4_/NiO nanocomposites are completely consistent with monoclinic BiVO_4_ (JCPDS No. 14-0688) and tetragonal SnO_2_ (JCPDS No. 46-1088) derived from FTO substrates. No other impurity phases were detected. The appearance of characteristic diffraction peaks at 2θ = 43.3° is corresponding to the (200) crystal plane of the cubic phase NiO, which can be concluded that the composite sample has been successfully prepared.

### Performance of the BiVO_4_/NiO Composite Photoanode

**FIGURE 3:**
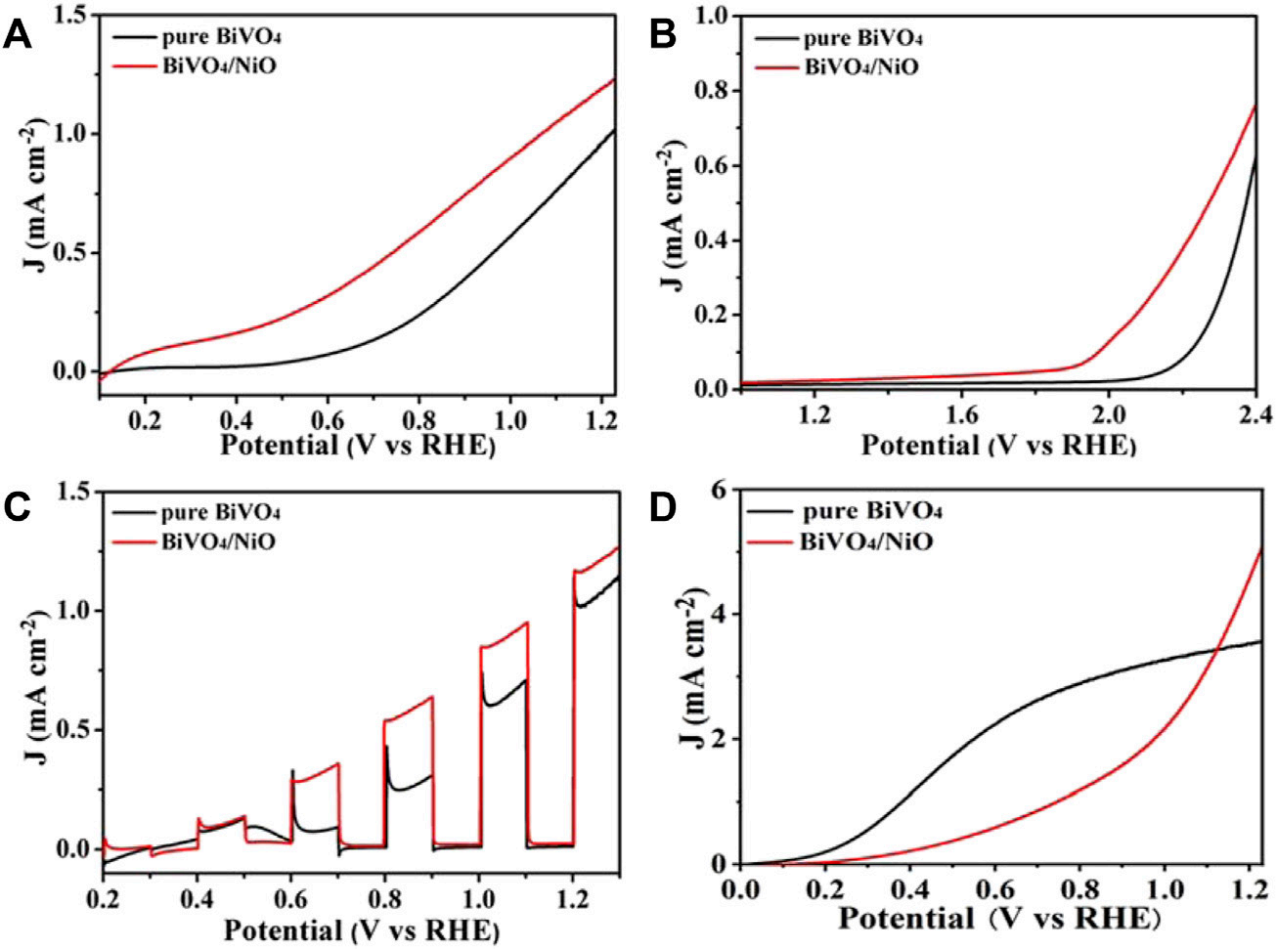
**(A)** Photocurrent density-voltage curve of BiVO_4_ and BiVO_4_/NiO photoanodes. **(B)** Photocurrent density-voltage curve of BiVO_4_ and BiVO_4_/NiO photoanodes in the absence of light. **(C)** Chopped linear sweep photocurrent-potential curve of BiVO_4_ and BiVO_4_/NiO. **(D)** Sulfite oxidation current curves.

In order to explore the effect of supported NiO on the PEC performance, the photoelectrochemical water splitting performance of BiVO_4_ photoanode modified by NiO cocatalyst was studied by electrochemical workstation. Linear sweep voltammetry (LSV) curves reflect the water oxidation properties of BiVO_4_/NiO and pure BiVO_4_. As shown in Figure 3A, the photocurrent density of unmodified BiVO_4_ at 1.23 V vs. RHE is 0.9 mA/cm^2^ and the onset potential is about 0.58 V vs. RHE, which is due to its unique structure and specific crystal orientation, leading to rapid transfer and separation of photogenerated carriers. After loading the NiO cocatalyst on BiVO_4_, the photoelectrode showed significant enhancement at all potentials, obtaining a photocurrent of 1.2 mA/cm^2^ at 1.23 V vs. RHE, which was higher than that of pristine BiVO_4_. In particular, BiVO_4_/NiO obtained a more negative onset potential compared to pure BiVO_4_, with a negative shift of about 0.35 V (Figure 3B). The increased photocurrent density and negatively shifted onset potential clearly indicate that the addition of NiO cocatalyst is a feasible way to enhance the water oxidation capacity of BiVO_4_ photoanode. Figure 3C shows the chopped photocurrent density−voltage (J−V) curves of BiVO_4_/NiO and pure BiVO_4_. All the photoanodes show an obvious “photo-switching” effect with fast response. Clearly, the BiVO_4_/NiO photoanode exhibits a much better PEC performance than BiVO_4_ film.

**FIGURE 4:**
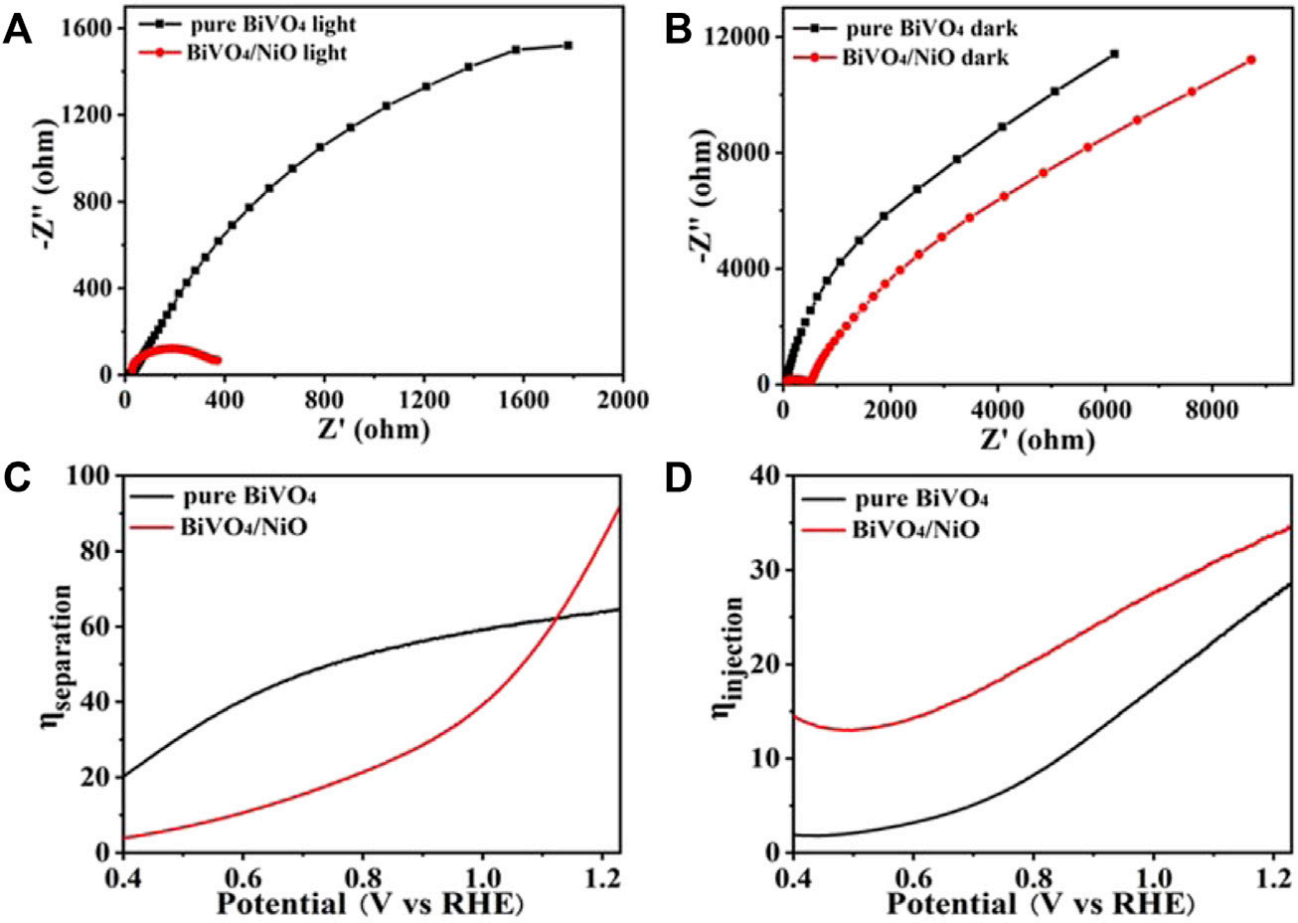
**(A)** EIS curves of pure BiVO_4_ and BiVO_4_/NiO under the light, **(B)** and in the dark. The EIS was measured at 1.23 V vs. RHE under an AM 1.5G solar simulator. **(C)** Charge separation efficiency versus potential curves and **(D)** charge injection efficiency versus potential curves of BiVO_4_, BiVO_4_/NiO.

The electron-hole pair recombination and charge generation kinetics of photoanode in PEC water oxidation process can be analyzed by EIS. The photoanode was measured at 1.23 V vs. RHE at AM 1.5G (100 mW/cm^2^), and the frequency range of the Nyquist plot was from 100 kHz to 0.1 Hz. The results of impedance spectra are useful for analyzing electrochemical surface reactions. The charge transfer resistance of the photoanode surface is estimated from the small semicircle in the Nyquist diagram, and the smaller the radius, the more effective the separation of charges. In addition, the EIS test result of dark reaction condition (Figure 4B) is consistent with that under light conditions in Figure 4A. The BiVO_4_/NiO photoanode exhibits the highest charge transportation, suggesting good charge separation ability.

To further explore the charge recombination at the BiVO_4_ and BiVO_4_/NiO photoanode interfaces, the charge separation and injection efficiencies were tested in Figures 4B,C. Charge separation efficiency is an important parameter to evaluate the proportion of carriers reaching the electrode surface/electrolyte interface to participate in water oxidation (Wang et al., 2017b). Therefore, the constant charge separation efficiency is shown in Figure 3D, ascribed to the photocurrent density of the BiVO_4_/NiO photoanode at an applied potential of 0.6 V vs. RHE when Na_2_SO_3_ was added to the electrolyte. The separation efficiency of the pure BiVO_4_ photoanode increases with the applied potential, especially it can reach about 60% at 1.23 V vs. RHE. However, the BiVO_4_/NiO nanostructured array photoanode exhibits a charge separation efficiency of 90% at 1.23 V vs. RHE. From this point of view, the cocatalyst NiO supported on BiVO_4_ can significantly improve the charge separation efficiency and facilitate the flow of charge carriers to the electrode surface/electrolyte interface to participate in water oxidation.

The photogenerated holes generated on the surface of the photoanode participate in the water oxidation reaction or recombine with electrons. The charge injection efficiency, defined as the fraction of those holes at the photoanode and electrolyte interface that is used for water oxidation reactions, can be improved by reducing surface recombination or accelerating hole transfer kinetics. (Zhou et al., 2020) As shown in Figure 4D, the charge injection efficiency of the pure BiVO_4_ photoanode reaches 28% 1.23 V vs. RHE. While the charge injection efficiency of the BiVO_4_/NiO photoanode increases to 35% in the potential range of 1.23 V vs. RHE.

**FIGURE 5:**
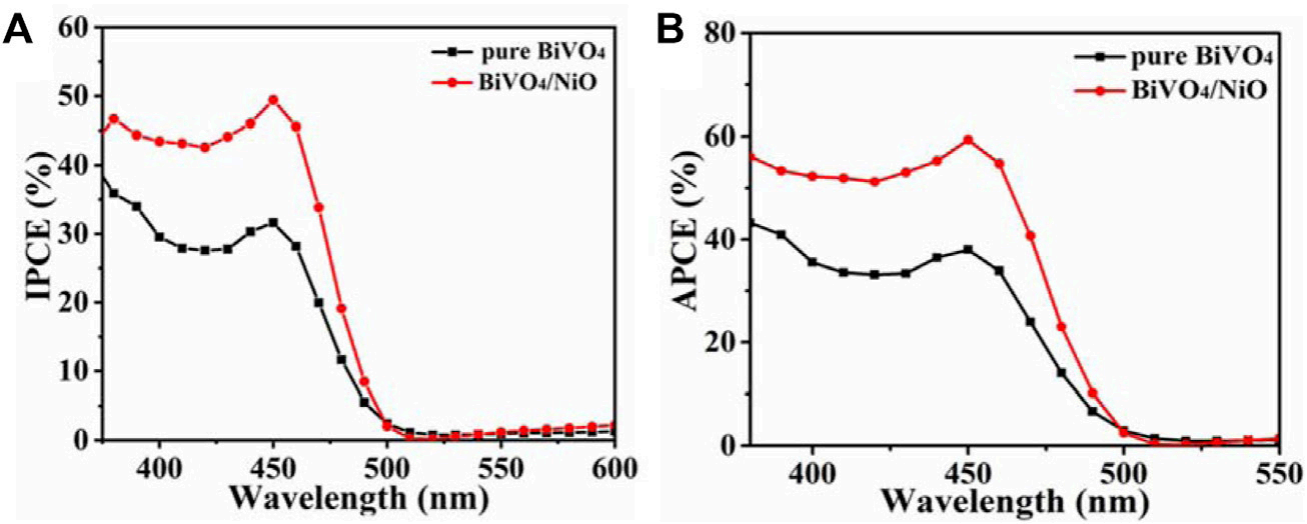
**(A)** IPCE of BiVO_4_, BiVO_4_/NiO measured at 1.23 V vs. RHE in the incident wavelength range from 380 to 600 nm. **(B)** APCE spectra for BiVO_4_, BiVO_4_/NiO photocathodes along with the AM 1.5 irradiance spectrum.

The quantum efficiencies of BiVO_4_, BiVO_4_/NiO photoanodes were determined by measuring incident photon current efficiency (IPCE) and absorbed photon current efficiency (APCE). (Wang et al., 2017b) The calculation of IPCE can refer to the following equation:

IPCE=(J×1240)/(P×λ)

where J is the current density (mA/cm^2^) measured at each specific wavelength, λ is the wavelength of the incident light (nm), and P is the power density of the incident light (mW/cm^2^). As shown in Figure 5A, BiVO_4_/NiO nanocomposites exhibit slightly higher IPCE values in the 450–500 nm range compared to bare BiVO_4_. The PEC performance mainly depends on the light-harvesting efficiency, charge separation efficiency, and collection yield. Since the light-harvesting efficiency is almost unchanged after the modification of the NiO cocatalyst, the enhancement of IPCE may be due to the fast separation of charge carriers and the accelerated water oxidation kinetics of the reaction, resulting in the enhanced photocurrent. The IPCE results are consistent with the above J-V measurements.

To obtain the absorbed photon-current efficiency, the APCE value of the photoanode was measured at 0.6 V vs. RHE, as shown in Figure 5B. The APCE value of BiVO_4_/NiO photoanode is significantly higher than that of BiVO_4_ from 380 to 500 nm, which is consistent with the overall PEC performance.

**FIGURE 6:**
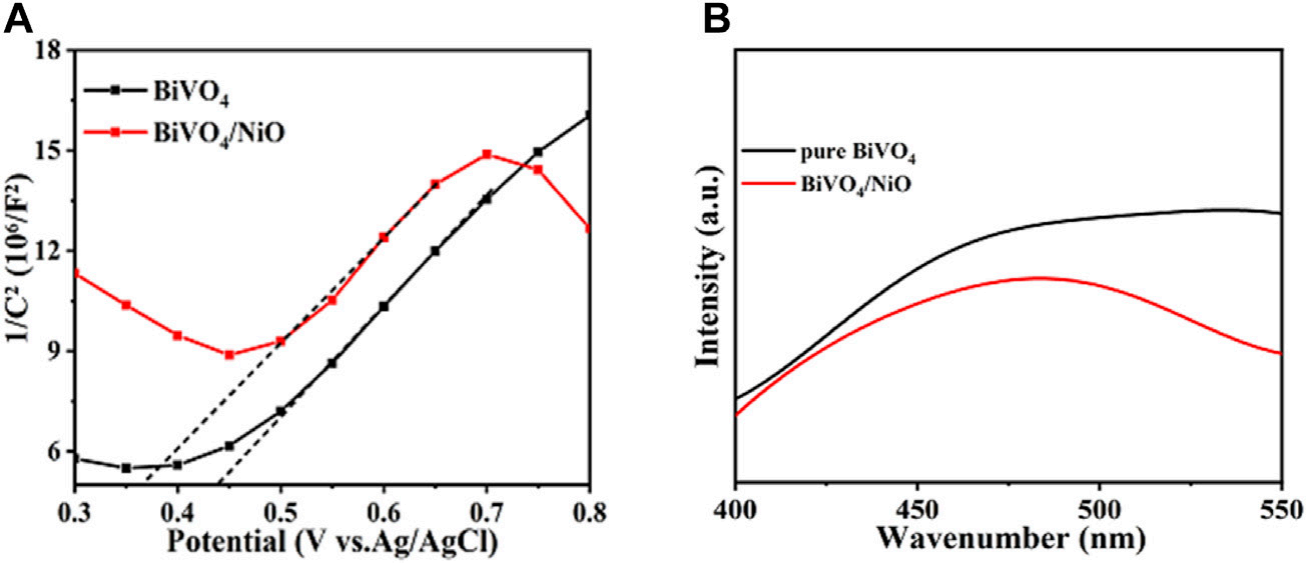
M-S diagram **(A)** and PL spectra of electrodes **(B)**.

Figure 6A shows the Mott Schottky barrier of BiVO_4_ and BiVO_4_/NiO. The capacitance-voltage curve is usually used to analyze the reasons for the enhancement of semiconductor performance. In order to better study the reasons for the increase of photocurrent after supporting the cocatalyst. Therefore, the capacitance-voltage curves of BiVO_4_ and BiVO_4_/NiO electrodes were measured under dark reaction conditions, and the *x*-axis tangent was made on the curves of BiVO_4_ and BiVO_4_/NiO. The tangent was positive, indicating that BiVO_4_ and BiVO_4_/NiO are both n-type semiconductors. The smaller slope of the composite electrode, the greater the carrier density. Thus, the BiVO_4_/NiO electrode has the largest carrier density. The increased carrier density causes the conductivity of BiVO_4_ to increase and ultimately increases its photocurrent density. Fluorescence spectroscopy (PL) can be used to effectively analyze the separation and recombination effects of photogenerated carriers. As shown in Figure 6B, it can be observed that the peak intensity of the BiVO_4_/NiO electrode is weaker than that of the pure BiVO_4_ electrode, which indicates that after NiO is loaded on the BiVO_4_ surface, the recombination rate of photo-generated electrons and holes becomes slower and the charge separation efficiency is improved.

**FIGURE 7:**
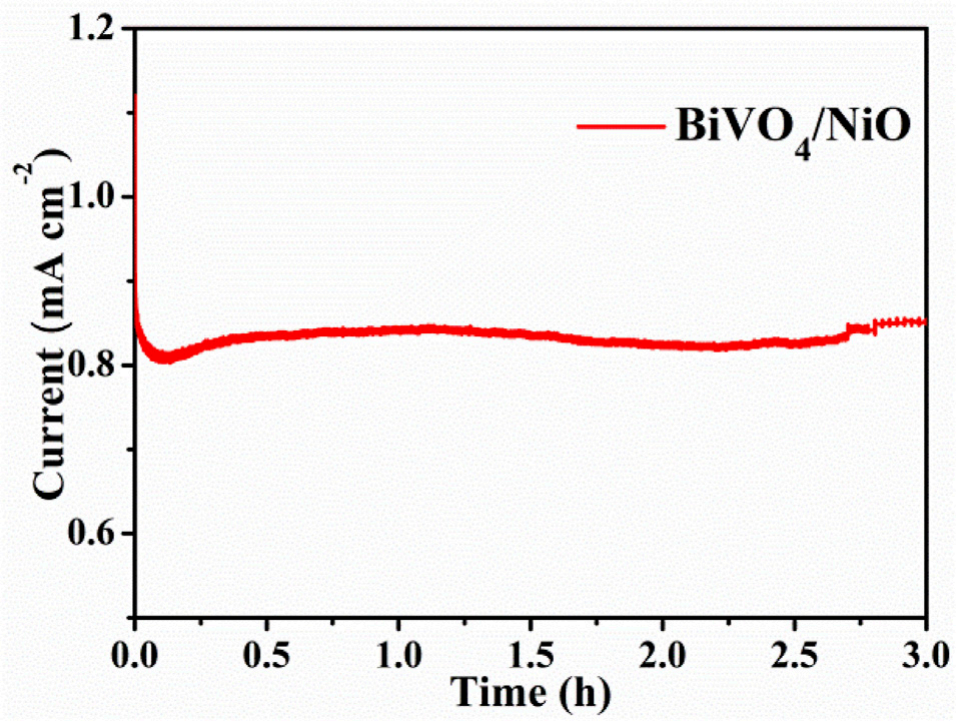
Stability curves of NiO/BiVO_4_ photoanode under the same conditions.

Stability test is an important index parameter to evaluate the effect of photoelectric catalyst and whether it has application value. The stability test of the BiVO_4_/NiO photoanode was analyzed under continuous irradiation under AM 1.5G. As shown in Figure 7, it can be observed that under the continuous irradiation of 3 h, the photocurrent density of the NiO/BiVO_4_ electrode is not significantly attenuated, indicating that the stability of the BiVO_4_ photoanode can be improved after loading NiO.

## Discussion on Mechanism

**FIGURE 8:**
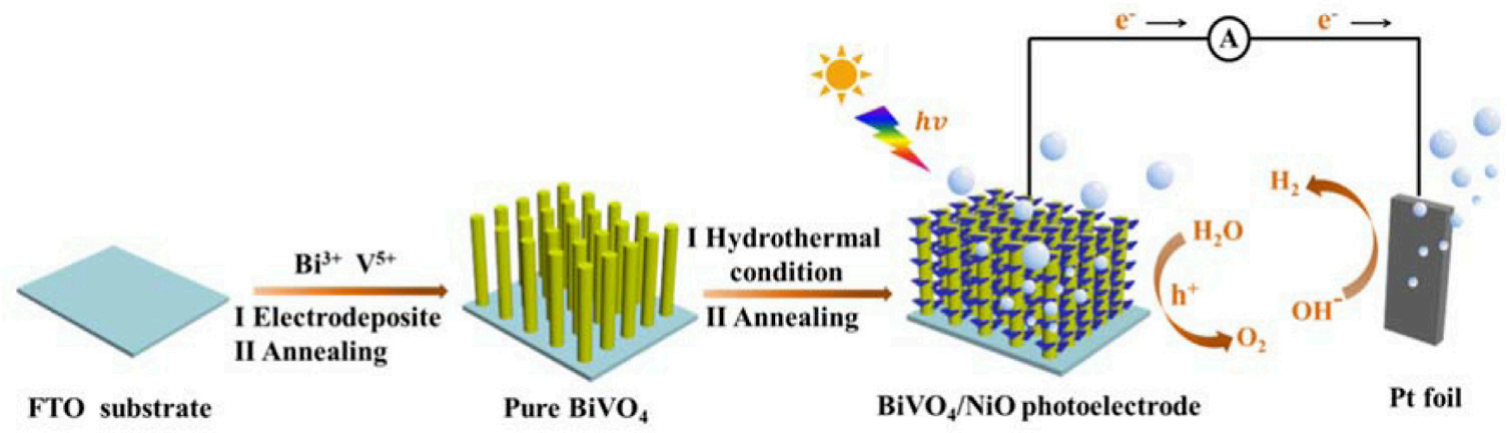
Schematic illustration of the fabrication process of BiVO_4_/NiO and the light harvesting and carrier separation mechanism in the BiVO_4_/NiO composite photoanode system.

Based on above results, the possible mechanisms for the enhancement in photoelectrocatalytic activity of BiVO_4_/NiO composite photoanode and the specific photogenerated charge carriers transfer are shown in Figure 8. In BiVO_4_ with monoclinic scheelite structure, the Bi 6s and O 2p orbits hybridize to form the valence band. When the BiVO_4_/NiO composite is irradiated with visible light, electron-hole pairs are generated in BiVO_4_, in which electrons in the valence band are excited to the conduction band and holes stay in the conduction band. With the NiO coated on the surface of BiVO_4_, NiO as a cocatalyst regulates the built-in electric field of BiVO_4_ photocatalyst, accelerates the charge separation rate of BiVO_4_, and thus the PEC performance of BiVO_4_ is improved.

## Conclusion

In conclusion, we successfully fabricated an efficient nanostructured BiVO_4_/NiO photoanode by a two-step method of hydrothermal calcination synthesis. The PEC performance of the NiO-modified BiVO_4_ photoanode was improved in 0.5 M Na_2_SO_4_ (pH = 6.86) electrolyte, reaching 1.2 mA/cm^2^ at 1.23 V vs. RHE, higher than that of the pure BiVO_4_ sample. In particular, the onset potential of the composite photoanode has a significant negative shift. The excellent PEC performance could be attributed to NiO abundant nano flake structure, the improved charge separation/transport efficiency and accelerated water oxidation kinetics thanks to the deposited NiO cocatalyst. Our work shed a light for design and fabrication of nanostructured photoelectrode with efficient PEC performances.

## Data Availability

The original contributions presented in the study are included in the article/Supplementary Material, further inquiries can be directed to the corresponding author.
